# A Worldwide Annotated Checklist of Fossil (Devonian–Cretaceous) Species of the Clam Shrimp Genus *Euestheria* (Branchiopoda: Diplostraca: Spinicaudata)

**DOI:** 10.3390/life14111438

**Published:** 2024-11-07

**Authors:** Shaohua Fang, Huanyu Liao

**Affiliations:** 1 Institute of Palaeontology, Yunnan Key Laboratory of Earth System Science, Yunnan Key Laboratory for Palaeobiology, MEC International Joint Laboratory for Palaeobiology and Palaeoenvironment, Yunnan University, Kunming 650500, China; 2 School of Earth Science, Yunnan University, Kunming 650500, China

**Keywords:** fossil record, arthropod, conchostracan, palaeogeographic distribution, stratigraphic distribution

## Abstract

Clam shrimps are one of the most common and representative invertebrates in continental strata and are endowed with important biostratigraphic and paleoecological values. The genus *Euestheria* is one of the most common clam shrimp taxa that has been recorded in the latest Paleozoic and the Mesozoic around the world. A list of all the species assigned to *Euestheria*, recorded from the Devonian to the Cretaceous across all seven continents as of September 2024, is provided. The list may serve as a valuable resource, potentially useful for the biostratigraphic division and long-range correlations of continental strata. In addition, the taxonomic issues of the genus are briefly introduced and examined herein. The study aims to provide a simple lead-in for all the researchers who have an interest in clam shrimp and the genus *Euestheria*.

## 1. Introduction

**Figure 1:**
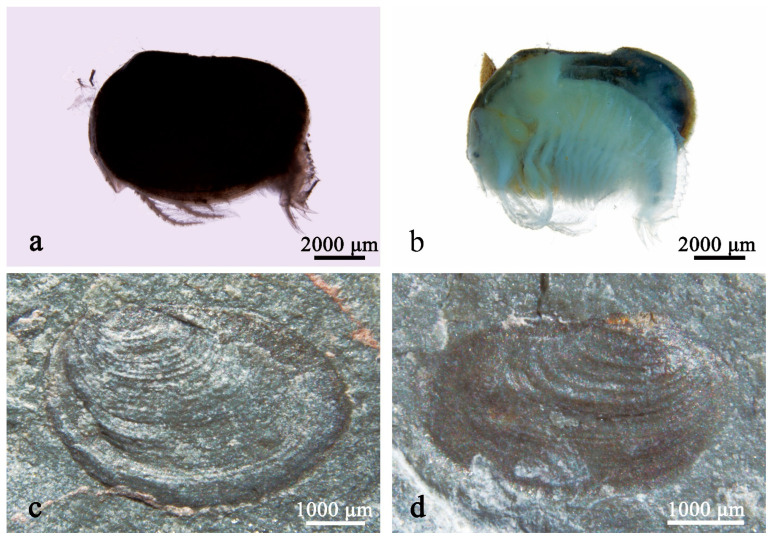
Modern and fossil specimens of clam shrimp: (**a**). a modern spinicaudatan with a whole carapace, from Tibet, China; (**b**). a modern spinicaudatan with the left valve of the carapace removed, showing “soft parts” of its body, from Tibet, China; (**c**). fossil specimen of *Euestheria* sp. from the Triassic Badong Formation in Hubei, China, showing the left valve of the carapace (housed at the Palaeobotanical Collections of the Institute of Palaeontology, Yunnan University, China, under catalog number YNUPB20001); (**d**). fossil specimen of *Euestheria* sp. from the Triassic Badong Formation in Hubei, China, showing the right valve of the carapace (housed at the Palaeobotanical Collections of the Institute of Palaeontology, Yunnan University, China, under catalog number YNUPB20002).

**Figure 2:**
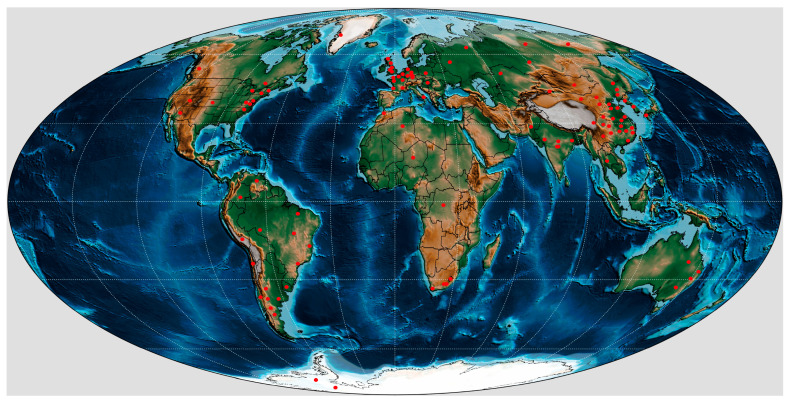
Global geographical distribution of all the *Euestheria* species records reviewed in the present paper shown on a map of present-day continental configurations (the map is based on Scotese [22]).

Clam shrimps, or “conchostracans,” are a paraphyletic group of bivalved crustaceans (Figure 1a–d) known in fossil records from the Paleozoic to recent times. Modern clam shrimps are usually seen in small lakes, ponds, swamps, temporary pools, and paddy fields with still water. Fossil clam shrimps are one of the most common and representative invertebrates in continental strata and are endowed with important biostratigraphic and paleoecological values [1,2,3,4,5,6,7,8]. The global fossil record indicates that the evolutionary history of these crustaceans can be at least traced back to the Early Devonian (Emsian) [8,9,10,11,12,13,14,15]. The genus *Euestheria* Depéret & Mazeran, 1912 is one of the most common taxa that has been recorded in the latest Paleozoic and the Mesozoic around the world (Figure 2) [2,4,5,11,16,17,18,19,20,21]. However, there is currently no comprehensive reference for the taxonomy and geographical and stratigraphic distributions of this “well-known” taxon. This study provides the first primary checklist of the global fossil record of the genus *Euestheria* based on an extensive literature survey, with a peculiar emphasis on their geographical and stratigraphic distributions. The checklist may serve as a valuable resource, potentially useful for the biostratigraphic division and long-range correlations of continental strata. Moreover, the taxonomic issues are briefly examined and discussed herein. All these works aim to provide a simple lead-in for all the researchers who have an interest in fossil clam shrimps and the genus *Euestheria*. 

## 2. Result 

### 2.1. Checklist

**Figure 3:**
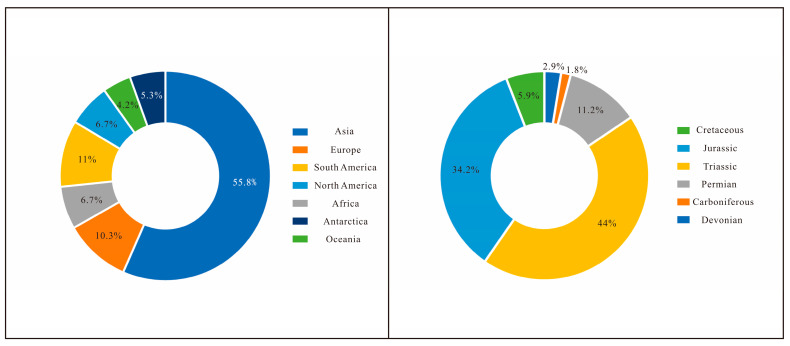
Percentage distribution of *Euestheria* species across modern continents (**left**) and across geological periods according to stratigraphic records (**right**).

Our study shows that a total of 249 species (217 named species including 10 new combinations and 32 names in open nomenclature (23 names with “conf.” and 9 with “aff.”)) have been assigned to *Euestheria*, formally recorded from the Devonian to the Cretaceous of all seven continents (Table 1). Two pie charts were made to show the percentages of species numbers geographically and chronologically (Figure 3). It is worth mentioning that our statistics and checklist may have several issues due to factors like the age of the publications, language barriers, and challenges in data collection caused by difficulties in accessing the literature. If readers notice any mistakes or missing information, we encourage them to reach out to us. 

### 2.2. Taxonomic Issues

Most fossil clam shrimps comprise carapaces only (Figure 1c,d). The important features of their crustacean body and appendages are often missing or poorly preserved in fossil specimens. As a result, the key characteristics of the body and appendages that are crucial in the morphological taxonomy of modern clam shrimps could not be used for morphological comparison between the fossil and modern species. The taxonomy of fossil clam shrimps is highly based on its carapace characteristics like the carapace size, shape, growth line numbers, growth band width, larval valve size, and particularly, the micro-ornamentations on the growth bands. The genus *Euestheria* is one of the most common taxa that has been recorded in all seven continents (Figure 3). Its type species *E. minuta* Zieten, 1833, from the Triassic of Germany [191], possesses an oval carapace ornamented with small polygonal reticulations (or irregular lirae) [9,21,117,191]. Observations of fossil clam shrimp in most early studies were performed under optical microscopy, and the micro-ornamentations, especially on those small, badly-preserved specimens, were not generally well recognized. As a result, numerous species with non-reticulate or unknown ornamentations were assigned to *Euestheria* according to their small oval carapace, fine growth lines, and small larval valve, making the genus a “dumping ground” of taxonomy. In recent years, with the widespread use of SEM, an increasing number of euestherids have been found to possess diverse non-reticulate ornamentations that are different from the reticulate ornamentations of the type species. For example, some Jurassic euestherids from China have been amended and reassigned to the genera *Qaidamestheria* Wang, 1983; *Tianzhuestheria* Shen, Li & Chen, 2002; and *Triglypta* Wang, 1984 [94,192,193,194,195,196,197], and their taxonomic status and descriptions have been changed. These genera possess punctae, punctae-formed reticulations, and linear arrangements or radial lirae on their growth bands [195,196]. Additionally, our recent studies have identified numerous Triassic “euestherids” that do not actually belong to the genus *Euestheria* (separate articles on this topic will be published). *Euestheria* is one of the most common taxa of fossil clam shrimps. However, serious taxonomic issues have impeded the use of this important taxon in global stratigraphic correlation. Hence, further studies on this taxon are desperately needed and will provide great help for comprehending both global stratigraphic correlation and the evolutionary history of clam shrimps.

## Figures and Tables

**Table 1:** Worldwide distributional and stratigraphical checklist of *Euestheria* species listed in chronological order based on the date of their description.

Species	Horizon, Age, and Locality	Reference
*Euestheria minuta* (Zieten, 1833)	Early–Middle Triassic, Europe (Germany and France); Lacoste borehole, Late Triassic, Gard, France; Vouhenans, borehole, Late Triassic, Haute-Saône, Vosges, France; Conliège borehole, Triassic, Jura, France; Triassic, Bex, Switzerland; Triassic, western High-Atlas, Morocco; Early Jurassic, Indre, France; Cape Biot Fm., Late Triassic, Greenland; Xujiahe Fm., Late Triassic, Sichuan, China; Ganhaizi Fm., Xiangyun Fm., Maichujing Fm., Manghuai Fm., Late Triassic, Yunnan, China; Feixianguan Fm., Yongningzhen Fm., Early Triassic, Guizhou, China; Yanchang Gr., Middle–Late Triassic, Gansu, China; Jiapeila Fm., Bagong Fm., Late Triassic, Tibet, China; Guanyintan Fm., Late Triassic, Hunan, China; Blizzard Heights Fm., Early Jurassic, Antarctica; Nanyinger Gr., Late Triassic, Gansu, China; Dingjiayao Fm., Middle Triassic, Gansu, China; Potrerillos Fm., Santa Clara de Abajo Fm., Middle–Late Triassic, Argentina; Wolfville Fm., Middle Triassic, Canada; Devonian, New York State, USA; Genkou Gr., Late Triassic, Guangdong, China; Tuckahoe Fm., Middle–Late Triassic, USA; Haojiagou Fm., Late Triassic, Xinjiang, China; Panchet Fm., Pali Fm., Late Permian–Early Triassic, Raniganj Coalfield, India; Santa Maria Fm., Middle Triassic, Brazil	Zieten, 1833; Defretin, 1953, 1969; Cuvelier et al., 2015; Katoo, 1971; Chen, 1974; Zhang et al., 1976; Luo et al., 2018; Shen, 1981; Gu & Cheng, 1981; Tasch, 1987; Cai, 1990, 1993; Gallego, 1992, 1999, 1999; Knox & Gordon, 1999; Zhang & Lin, 2000; Kozur & Weems, 2007, 2010; Sun et al., 2010; Ghosh, 2011; Jenisch et al., 2017. [2,4,5,14,18,19,23,24,25,26,27,28,29,30,31,32,33,34,35,36,37,38]
*Euestheria* cf. *minuta*	Tongchuan Fm., Middle Triassic, Shaanxi, China; Xingkoushi Fm., Early Jurassic, Hebei, China; Blina Shale Fm., Early Triassic, Australia; Cacheuta Fm., Middle–Late Triassic, Argentina; Potrerillos Fm., Late Triassic, Argentina	Wang & Liu, 1980; Shen & Chang, 1993; Tasch, 1987; Gallego, 1999, 1999; Tassi et al., 2015. [19,32,33,39,40,41]
*Euestheria* aff. *minuta*	Santa Maria Fm., Middle Triassic, Brazil	Katoo, 1971. [26]
*Euestheria albertii* (Voltz, 1835)	Moenkopi Fm., Middle Triassic, Arizona, USA	Voltz, 1835; Lucas & Schoch, 2002; Kozur & Weems, 2010. [4,42,43]
*Euestheria albertii mahlerselli* Kozur & Lepper	Solling Fm, Early Triassic, Franconia, Bavaria, Germany	Kozur & Mock, 1993. [44]
*Euestheria vatus* (Lea, 1856)	Triassic, USA	Lea, 1856; Spamer, 1989. [45,46]
*Euestheria brodieana* (Jones, 1862)	Triassic, Gloucestershire, Warwickshire, England; Yanchang Gr., Late Triassic, Shaanxi, China; Yanchang Fm., Late Triassic, Shaanxi, China; Midland Fm., Late Triassic, England; Waterfall Fm., Early Jurassic, France, Germany; Late Triassic, USA	Jones, 1862; Wang & Liu, 1980; Pang, 1993; Kozur & Weems, 2007, 2011. [36,39,47,48,49]
*Euestheria middendorfii* (Jones, 1862)	Jurassic, Turgay Plateau, Kazakhstan	Jones, 1862. [47]
*Euestheria mangaliensis* (Jones, 1862)	Triassic, India; Early Triassic, Wardha Basin, India; Early Triassic, Nágpur. India; Argentina; Sunjiagou Fm., Permian, Shaanxi, China; Kamthi Fm., Early Triassic, Berwickshire, England	Jones, 1862; Tasch, 1987; Gallego, 1992; Liu & He, 2000; Ghosh, 2011. [5,19,31,47,50]
*Euestheria kotnhensis* (Jones, 1862)	Early Jurassic, India; Middle Triassic, Kota, India	Jones, 1862; Kobayashi, 1954. [16,47]
*Euestheria forbesi* (Jones, 1862)	Mesozoic, South America; Late Triassic, Mendoza, Argentina; Ischichuca Fm., Middle–Late Triassic, La Rioja, Argentina	Jones, 1862; Tasch, 1987; Gallego et al., 2001. [19,47,51]
*Euestheria* cf. *forbesi*	Santa Maria Fm., Middle Triassic, Brazil	Gallego, 1996. [52]
*Euestheria murchisoniae* (Jones, 1862)	Late Jurassic, Scotland	Jones, 1862; Kobayashi, 1954. [16,47]
*Euestheria orientalis* Eichwald, 1868	Jurassic, Nertschinsk, Russia	Eichwald, 1868; Kobayashi, 1954. [16,53]
*Euestheria dawsoni* (Jones, 1870)	Canso Gr., Carboniferous, New Hampshire, USA; Cheverie Fm., Canada; Anzin Fm., Flines Fm., Carboniferous, Pas-de-Calais, France	Jones,1870; Copeland, 1957; Bell, 1960; Cuvelier et al., 2015. [14,54,55,56]
*Euestheria rimosa* (Goldenberg, 1877)	Steinkohlen Fm., Permian, Saarland, Germany	Goldenberg, 1877. [57]
*Euestheria limbata* (Goldenberg, 1877)	Steinkohlen Fm., Permian, Saarland, Germany	Goldenberg, 1877. [57]
*Euestheria greyi* (Jones, 1878)	Late Permian, Cape Town, South Africa	Jones, 1878; Tasch, 1987. [19,58]
*Euestheria karpinskiana* (Jones, 1883)	Triassic, Siberia, Russia	Jones, 1883; Kobayashi, 1954. [16,59]
*Euestheria laxitecta* (Jones, 1890)	Triassic, Gipskeuper, Germany; Late Triassic, England; Late Triassic, Krasiejów, Poland.	Jones, 1890; Olempska, 2004. [60,61]
*Euestheria* ? *laitexta*	Xujiahe Fm., Late Triassic, Chongqing, China	Chen, 1974; Zhang et al., 1976. [2,18]
*Euestheria cebnnensis* (Grand & Eury, 1890)	Lower Stephanian, Carboniferous, La Mure Basin, France	Grand, 1890. [62]
*Euestheria anomala* (Jones, 1901)	Early Cretaceous, Heidelberg, Swellendam, South Africa	Jones, 1901; Tasch, 1987. [19,63]
*Euestheria simoni* Pruvost, 1911	Bruay Fm., Westphalian C (now late Moscovian), Carboniferous, Nord, France; Rabat, Morocco; South Wales, England	Pruvost, 1911; Cuvelier et al., 2015; Dix & Trueman, 1928; Tasch, 1987. [14,19,64,65,66]
*Euestheria kawasakii* Ozawa & Watanabe, 1923	Mesozoic, Korea; Xiangyun Fm., Late Triassic, Yunnan, China; Amison Fm., Late Triassic, South Korea	Ozawa & Watanabe, 1923; Zhang et al., 1976; Kim & Lee, 2015. [2,67,68]
*Euestheria elongata* Tchernychev, 1926	Permian, Russia	Tchernychev, 1926. [69]
*Euestheria* ? *elongata*	Baitianba Fm., Early Jurassic, Sichuan, China	Zhang et al., 1976. [2]
*Euestheria novocastrensis* Mitchell, 1927	Newcastle Coal Measures, Dirty Coal seam, Late Permian, New South Wales, Australia	Mitchell, 1927; Tasch, 1987. [19,70]
*Euestheria ipsviciensis* Mitchell, 1927	Late Triassic, Denmark Hill, Ipswich, Queensland, Australia	Mitchell, 1927; Tasch, 1987. [19,70]
*Euestheria belmontensis* Mitchell, 1927	Late Permian, Dirty Coal seam, New South Wales, Australia	Mitchell, 1927; Tasch, 1987. [19,70]
*Euestheria trigonellaris* Mitchell, 1927	Late Permian, Dirty Coal seam, New South Wales, Australia	Mitchell, 1927; Kobayashi, 1954; Tasch, 1987. [16,19,70]
*Euestheria nyuicastrensis* Mitchell, 1927	Late Permian, New South Wales, Australia	Mitchell, 1927; Kobayashi, 1954. [16,70]
*Euestheria obliqua* Mitchell, 1927	Late Permian, New South Wales, Australia	Mitchell, 1927; Kobayashi, 1954. [16,70]
*Euestheria dahurica* Chernyshev, 1930	Early Cretaceous, Russia	Chernyshev, 1930. [71]
*Euestheria* cf. *dahurica*	Early Cretaceous, Africa	Gallego et al., 2020. [6]
*Euestheria nengkiangensis* Chi, 1931	Nenjiang Fm., Early Cretaceous, Heilongjiang, China	Ji, 1931. [72]
*Euestheria sinkiangensis* Chi, 1931	Late Jurassic, Turpan, Xinjiang, China; Sangonghe Fm., Early Jurassic, Xinjiang, China	Ji, 1931; Zhang et al., 1976; Wei, 1984. [2,72,73]
*Pseudestheria diensti* Poschmann, 2024 (=*Euestheria diensti* Gross, 1934)	Devonian, Germany	Gross,1934; Poschmann et al., 2024 [3,74]
*Euestheria gutta* (Lyutkevich, 1937)	Calvrd Fm., Early Triassic, Germanic Basin, Germany; Jaworznia Fm., Late Permian, Holy Cross Mountains, Poland; Sunjiagou Fm., Late Permian, Shaanxi, China; Kayitou Fm., Early Triassic, Guizhou, China; Vokhma Fm., Obnora Fm., Early Triassic, Russia; Charkabozhskaya Fm., Early Triassic, Myla Rive, Komi Republic, Russia	Lvutkeyich, 1937; Kozur & Seidel, 1983; Ptaszyński & Niedźwiedzki, 2004; Chu et al., 2013; Chu et al., 2018; Scholze et al., 2020; Miao et al., 2021; Scholze et al., 2015, 2017; Vladimirovna et al., 2020. [15,75,76,77,78,79,80,81,82,83]
*Euestheria* aff. *gutta*	Kayitou Fm., Early Triassic, Guizhou, China	Scholze et al., 2020. [80]
*Euestheria aequale* (Lyutkevich, 1937)	Permian, Russia	Lvutkeyich, 1937. [75]
*Euestheria evenkiensis* (Lyutkevich, 1937)	Late Permian, Late Tatarian (now Changhsingian), Central Siberia, Russia	Lvutkeyich, 1937; Cuvelier et al., 2015. [14,75]
*Euestheria hausmanni* Schmidt, 1938	Cumnock Fm., Lockatong Fm., New Oxford Fm., Late Triassic, Coburg Sandstein, Germany	Schmidt, 1928; Kozur & Weems, 2007. [36,84]
*Euestheria stockmansi* Maillieux, 1939	Early Devonian, Belgium; Devonian, Vincy, France; Early Triassic, Australia	Maillieux, 1939; Raymond, 1946; Kobayashi, 1954; Defretin, 1950; Novojilov, 1958. [10,11,16,85,86]
*Euestheria subsmoni* Deleau, 1945	Early Carboniferous, Algeria	Deleau, 1945. [87]
*Euestheria exsecta* Novojilov, 1946	Triassic, Russia; Volpriehausen Fm. and Detfurth Fm., Early Triassic, Germanic Basin, Germany; Hardegsen Fm., Early Triassic, Siberia, Russia; Csopak Marl Fm., Early Triassic, Hungary	Novojilov, 1946; Lucas & Schoch, 2002; Kozur & Weems, 2010. [1,4,43]
*Euestheria multinstila* Novojilov, 1946	Permian, Russia	Novojilov, 1946; Kobayashi, 1954. [1,16]
*Euestheria belli* Raymond, 1946	Cheverie Fm., Richmond County, Canada	Raymond, 1946; Bell, 1960. [11,56]
*Euestheria autunensis* Raymod, 1946	Permian, France	Raymond, 1946. [11]
*Euestheria anchietai* Teixeira, 1947	Late Permian, Africa	Teixeira, 1947. [88]
*Euestheria striolatissima* Rusconi, 1948	Middle Triassic, Argentina	Rusconi, 1948. [89]
*Euestheria lerichei* Marliere, 1950	Late Cretaceous, Africa	Mouta & Marlière, 1950. [90]
*Euestheria passaui* Marliere, 1950	Jurassic, Congo	Mouta & Marlière, 1950; Egoroff & Lombard, 1961. [90,91]
*Euestheria ricourr* Defretin, 1950	Triassic, France	Defretin, 1950. [12]
*Euestheria mendesi* Almerida, 1950	São Paulo, Brazil	Almerida, 1950. [92]
*Euestheria khinganensis* Kobayashi, 1951	Jurassic, Jilin, China	Kobayashi, 1951; Zhang et al., 1976. [2,93]
*Euestheria rampoensis* Kobayashi, 1951	Gabisan Fm., South Korea; Amisan Fm., Late Triassic, South Korea	Kobayashi, 1951; Kim & Lee, 2015. [68,93]
*Euestheria* aff. *rampoensis*	Middle Jurassic, Hebei, China	Wang et al., 1984. [94]
*Euestheria shimamurai* Kobayashi, 1951	Late Triassic, North Korea; Xiangyun Fm., Late Triassic, Yunnan, China; Amison Fm., Late Triassic, South Korea	Kobayashi, 1951; Kobayashi, 1954; Zhang et al., 1976; Kim & Lee, 2015. [2,16,68,93]
*Euestheria* cf. *shimamurai*	Nanyinger Gr., Late Triassic, Gansu, China	Kobayashi, 1951; Zhang et al., 1976. [2,93]
*Euestheria atsuensis* Kobayashi, 1951	Triassic, Japan	Kobayashi, 1951. [93]
*Euestheria* ? cf. *atsuensis*	Xujiahe Fm., Late Triassic, Sichuan, China	Zhang et al., 1976. [2]
*Congestheriella olsoni* Bock, 1954 (=*Euestheria olsoni* Bock, 1953)	La Quinta Fm., Late Triassic or Early Jurassic, Andean Cordillera, Venezuela	Bock, 1953; Gallego et al., 2010. [95,96]
*Euestheria princetonensis* Bock, 1953	Lockatong Fm., Late Triassic, New Jersey, USA	Bock, 1953. [95]
*Euestheria winterpockensis* Bock, 1953	Late Triassic, Virginia, USA; Grabfeld Fm., Tuckahoe Fm., and Falling Creek Fm., Late Triassic, Germany; Passaic Fm. and Bull Run Fm., Late Triassic, New Jersey, USA; Bocas Fm. and Montebel Fm., Late Triassic, Colombia	Bock, 1953; Kozur & Weems, 2007; Weems & Lucas, 2015; Alarcón et al., 2024. [36,95,97,98]
*Euestheria eifelensis* Novojilov, 1953 (=*Palaeolimnadiopsis eifelensis* Raymond, 1946)	Early Devonian, Germany; Devonian, Russia	Raymond, 1946; Novojilov, 1953. [11,99]
*Euestheria chop rensis* Novojilov, 1953	Devonian, Russia	Novojilov, 1953. [99]
*Euestheria sainsllalidensis* Novojilov, 1953	Mongolia	Novojilov, 1953. [99]
*Euestheria* aff. *sainsllalidensis*	Jurassic, Russia	Stepanov, 1966. [100]
*Euestheria kidoi* Kobayashi, 1954	Late Triassic, Korea	Kobayashi, 1954. [16]
*Euestheria kusumi* Kobayashi, 1954	Late Triassic, Korea	Kobayashi, 1954. [16]
*Euestheria langei* Mendes, 1954	Permian, Brazil	Mendes, 1954. [101]
*Euestheria janovi* Novojilov, 1955	Devonian, Russia	Novojilov, 1955. [102]
*Euestheria consummata* Novojilov, 1955	Devonian, Russia	Novojilov, 1955. [102]
*Euestheria ltakassica* Novojilov, 1955	Devonian, Russia	Novojilov, 1955. [103]
*Euestheria leonardii* Tasch, 1958 (=*Euestheria harveyi wellingtoni* Tasch, 1956)	Permian, Kansas, USA	Tasch, 1956, 1958. [104,105]
*Euestheria leonardii* Tasch, 1958 (=*Euestheria harveyi harveyi* Tasch, 1956)	Permian, Kansas, USA	Tasch, 1956, 1958. [104,105]
*Euestheria leonardii* Tasch, 1956	Permian, Kansas, USA	Tasch, 1956, 1958. [104,105]
*Euestheria lamberti* Defretin, 1956	Agades, Africa	Defretin et al., 1956; Defretin, 1958. [106,107]
*Euestheria marginata* Defretin, 1956	Agades, Africa	Defretin et al., 1956; Defretin, 1958. [106,107]
*Euestheria azambujai* Pinto, 1956	Santa Maria Fm., Late Triassic, Rio Grande do Sul, Brazil	Pinto, 1956. [108]
*Euestheria raymondi* Copeland, 1957	Canso Gr., Early Mississippian (Carboniferous), Canada	Copeland, 1957. [55]
*Euestheria jakutica* Kashirtsev, 1957	Early Triassic, Russia	Kashirtsev, 1957. [109]
*Euestheria kaschirzwi* Kashirtsev, 1957	Early Triassic, Russia	Kashirtsev, 1957. [109]
*Euestheria lirella* Bell, 1960	Cheverie Fm., Horton Gr., Canada	Bell, 1960. [56]
*Euestheria* cf. *lirella*	Albert Fm., New Brunswick, Canada	Greiner, 1974. [110]
*Euestheria kokurensis* Kusumi, 1960	Cretaceous, Kokura, Japan	Kusumi, 1960. [111]
*Euestheria imamurai* Kusumi, 1960	Cretaceous, Kokura, Japan	Kusumi, 1960. [111]
*Euestheria taniiformis* Zaspelova, 1961	Sangonghe Fm., Early Jurassic, Xingjiang, China; Wennan Fm., Early Jurassic, Shandong, China.	Zaspelova, 1961; Lu, 1995; Chen, 1982. [112,113,114]
*Euestheria* cf. *taniiformis*	Xingkoushi Fm., Early Jurassic, Hebei, China	Shen & Chang, 1993. [40]
*Euestheria tenuiformis* Zaspelova 1961	Sangonghe Fm., Early Jurassic, Xinjiang, China	Zaspelova, 1961; Chen, 1995. [112,115]
*Euestheria spitzbergensis* Zaspelova 1961	Late Triassic, Russia	Zaspelova, 1961. [112]
*Euestheria* ? *taniiformis*	Baitianba Fm., Shiwandashan Gr., Early Jurassic, Sichuan, Guangxi, China	Zhang et al., 1976. [2]
*Euestheria* aff. *taniiformis*	Badaowan Fm., Early Jurassic, Xinjiang, China	Chen, 2003. [116]
*Euestheria franconica* (Reible, 1962)	Meißner Fm., Middle Triassic, USA	Reible, 1962; Sell, 2018. [117,118]
*Euestheria* cf. *franconica*	Erfurt Fm., Middle Triassic, USA	Sell, 2018. [118]
*Euestheria elliptica* Molin, 1965	Permian, Russia; Ermaying Fm., Middle Triassic, Shaanxi, China	Molin, 1965; Wu, 1991. [119,120]
*Euestheria osvanjensis* Molin, 1965	Early Triassic, Russia	Molin, 1965. [119]
*Euestheria udorica* Molin, 1966	Early Triassic, Russia	Molin, 1966; Lipatova & Lopato, 2000. [121,122]
*Euestheria sambaensis* Simone, 1967	Cretaceous, Congo	Defretin, 1967. [123]
*Euestheria sambaensis* Defretin & Lefranc, 1967	Early Cretaceous, Africa	Defretin, 1967. [123]
*Euestheria bourozi* Defretin & Lefranc, 1970	Bruay Fm., Westphalian (now late Moscovian), Carboniferous, Pas-de-Calais, France	Cuvelier et al., 2015. [14]
*Euestheria volkheimeri* Tasch, 1970	Canadon Asfalto Fm., Jurassic, Patagonia, South America	Tasch, 1970. [124]
*Euestheria syntchaense* Novojilov, 1970	Late Triassic, Russia	Novojilov, 1970; Chunikhin, 2009. [125,126]
*Euestheria spltzbergensis* Zaspelova, 1972	Late Triassic, Russia	Zaspelova et al., 1972. [127]
*Euestheria tumida* Zaspelova, 1972	Late Triassic, Russia	Zaspelova et al., 1972. [127]
*Euestherie* ? *luchangensis* Chen, 1974	Baiguowan Fm., Late Triassic, Sichuan, China	Chen, 1974; Zhang et al., 1976. [2,18]
*Euestheria yimengensis* Chen, 1974	Baiguowan Fm., Late Triassic, Sichuan, China	Chen, 1974; Zhang et al., 1976. [2,18]
*Euestheria contracta* Chen, 1974	Baiguowan Fm., Late Triassic, Sichuan, China	Chen, 1974; Zhang et al., 1976. [2,18]
*Euestheria weiyuanensis* Chen, 1974	Xujiahe Fm., Late Triassic, Chongqing, China	Chen, 1974; Zhang et al., 1976. [2,18]
*Euestheria dazuensis* Chen, 1974	Xujiahe Fm., Late Triassic, Chongqing, China; Baiguowan Fm., Late Triassic, Sichuan, China; Ganhaizi Fm. and Xiangyun Fm., Late Triassic, Yunnan, China; Manghuai Fm., Middle Triassic, Yunnan, China	Chen, 1974; Luo et al., 2018. [18,27]
*Euestheria yipinglangensis* Chen, 1974	Xujiahe Fm., Late Triassic, Chongqing, China; Baiguowan Fm., Late Triassic, Sichuan, China; Ganhaizi Fm. and Xiangyun Fm., Late Triassic, Yunnan, China; Yanchang Gr., Late Triassic, Ningxia, China; Bagong Fm., Late Triassic, Tibet, China; Dingjiayao Fm., Middle Triassic, Gansu, China; Genkou Gr., Late Triassic, Guangdong, China; Manghuai Fm., Middle Triassic, Yunnan, China	Chen, 1974; Zhang et al., 1976; Chen & Shen, 1981; Cai, 1990; Zhang & Lin, 2000; Luo et al., 2018. [2,18,27,29,35,128]
*Euestheria* cf. *yipinglangensis*	Xingshikou Fm., Early Jurassic, Hebei, China	Shen & Chang, 1993. [40]
*Euestheria hechuanensis* Chen, 1974	Xujiahe Fm., Late Triassic, Chongqing, China	Chen, 1974; Zhang et al., 1976. [2,18]
*Euestheria* ? *tianquanensis* Chen, 1974	Xujiahe Fm., Late Triassic, Sichuan, China	Chen, 1974; Zhang et al., 1976. [2,18]
*Euestheria buravasi* Kobayashi, 1975	Nam Pha Fm. and Huai Hin Lat Fm., Late Triassic, Khorat Plateau, Thailand; Cumnock Fm. and Catharpin Creek Fm., Late Triassic, USA; Sanford Fm., Late Triassic, USA	Kobayashi, 1975; Kozur & Weems, 2007, 2010; Chitnarin et al., 2022. [4,36,129,130]
*Euestheria mansuyi* Kobayashi, 1975	Nam Pha Fm., Late Triassic, Nam Phrom Dam, Thailand	Kobayashi, 1975; Chonglakmani et al., 1990. [129,131]
*Euestheria thailandica* Kobayashi, 1975	Nam Pha Fm., Late Triassic, Nam Phrom Dam, Thailand	Kobayashi, 1975. [129]
*Euestheria bunopasi* (=*Cyclestherioides bunopasi* Kobayashi, 1975)	Nam Pha Fm., Late Triassic, Nam Phrom Dam, Thailand	Kobayashi, 1975, 1984; Burrett, 2021. [129,132,133]
*Euestheria rhombica* Chen, 1976	Yanchang Gr., Late Triassic, Gansu, China; Genkou Gr., Late Triassic, Guangdong, China	Zhang et al., 1976; Zhang & Lin, 2000. [2,35]
*Euestheria mupangensis* Chen, 1976	Xiangyun Fm. and Maichujing Fm., Late Triassic, Yunnan, China; Bagong Fm. and Jiapeila Fm., Tibet, China; Wenbinshan Fm., Late Triassic, Fujian, China	Zhang et al., 1976; Lv et al., 2016; Chen & Shen, 1981; Liang, 1998. [2,128,134,135]
*Euestheria obliqua* Chen, 1976	Xiangyun Fm., Late Triassic, Yunnan, China; Dirty Coal Seam, Late Permian, New South Wales, Australia; Genkou Gr., Late Triassic, Guangdong, China	Zhang et al., 1976; Tasch, 1987; Zhang & Lin, 2000. [2,19,35]
*Euestheria angusta* Chen, 1976	Xiangyun Fm., Late Triassic, Yunnan, China; Bagong Fm., Late Triassic, Tibet, China	Zhang et al., 1976; Chen & Shen, 1981. [2,128]
*Euestheria* cf. *angusta*	Xingshikou Fm., Early Jurassic, Hebei, China	Shen & Chang, 1993. [40]
*Euestheria lata* Chen, 1976	Ganhaizi Fm., Late Triassic, Yunnan, China; Bagong Fm., Late Triassic, Tibet, China; Dudley seam, Dirty Coal seam, Permian, Niger, Australia	Zhang et al., 1976; Chen & Shen, 1981; Tasch, 1987. [2,19,128]
*Euestheria xiangyunensis* Chen, 1976	Xiangyun Fm. and Maichujin Fm., Late Triassic, Yunnan, China	Zhang et al., 1976; Lv et al., 2016. [2,134]
*Euestheria yanjingxiensis* Shen, 1976	Xujiahe Fm., Late Triassic, Chongqing, China; Kayitou Fm., Early Triassic, Guizhou, China; Zhangping Fm., Middle Jurassic, Fujian, China	Zhang et al., 1976; Chu et al., 2013; Cao, 1989. [2,78,136]
*Euestheria langdaiensis* Shen, 1976	Feixianguan Fm., Early Triassic, Guizhou, China	Zhang et al., 1976. [2]
*Euestheria langdaiensis magna*	Bernburg Fm., Early Triassic, Germany	Kozur, 1980. [137]
*Euestheria orbicula* Shen, 1976	Yongningzhen Fm., Kayitou Fm., Early Triassic, Guizhou, China	Zhang et al., 1976; Chu et al., 2013. [2,78]
*Euestheria leidaiyanensis* Shen, 1976	Yongningzhen Fm., Early Triassic, Guizhou, China	Zhang et al., 1976. [2]
*Euestheria hubeiensis* Shen, 1976	Badong Fm., Middle Triassic, Hubei, China	Zhang et al., 1976. [2]
*Euestheria* cf. *hubeiensis*	Xujiashan Fm., Middle Triassic, Jiangsu, China	Min et al., 1981. [138]
*Euestheria lepida* Shen, 1976	Badong Fm., Middle Triassic, Hubei, China	Zhang et al., 1976. [2]
*Euestheria dactylis* Shen, 1976	Badong Fm., Middle Triassic, Hubei, China; Kayitou Fm., Early Triassic, Guizhou, China	Zhang et al., 1976; Chu et al., 2013. [2,78]
*Euestheria multilinearis* (Shen, 1976)	Badong Fm., Middle Triassic, Hubei, China	Zhang et al., 1976. [2]
*Euestheria shizibaoensis* Shen, 1976	Badong Fm., Middle Triassic, Hubei, China; Kayitou Fm., Early Triassic, Guizhou, China	Zhang et al., 1976; Chu et al., 2013. [2,78]
*Euestheria* cf. *shizibaoensis*	Xujiashan Fm., Middle Triassic, Jiangsu, China	Min et al., 1981. [138]
*Euestheria sparsa* Shen, 1976	Badong Fm., Middle Triassic, Hubei, China	Zhang et al., 1976. [2]
*Triglypta shandanensis* Liao, 2017 (=*Euestheria shandanensis* Chen, 1976)	Longfengshan Gr., Early–Middle Jurassic, Gansu, China; Wangjiashan Fm., Middle Jurassic, Gansu, China; Lishan Fm., Early Jurassic, China; Dongyingfang Fm., Middle Jurassic, Liaoning, China; Wenbinshan Fm., Late Triassic, Fujian, China; Xiaoping Fm., Late Triassic, Guangdong, China; Sangonghe Fm., Badaowan Fm., Early Jurassic, Xinjiang, China	Zhang et al., 1976; Li et al., 1982; Cao, 1989; Li, 1990; Liang, 1998; Zhang & Lin, 2000; Shen, 2003. [2,35,136,139,140,141]
*Euestheria* aff. *shandanensis*	Wennan Fm., Early Jurassic, Shandong, China	Chen, 1982. [114]
*Euestheria* cf. *shandanensis*	Xingshikou Fm. and Laohugou Fm., Early Jurassic, Hebei, Liaoning, China; Sangonghe Fm., Early Jurassic, Xinjiang, China	Shen & Chang, 1993; Fu, 1998. [40,142]
*Euestheria ziliujingensis* Chen, 1976	Haifanggou Fm., Middle Jurassic, Liaoning, China; Shaximiao Fm., Middle Jurassic, Guizhou, China; Xiashaximiao Fm., Middle Jurassic, Sichuan, Chongqing, China; Luoao Fm., Middle Jurassic, Jiangxi, China; Jiulongshan Fm., Middle Jurassic, Hebei, China; Zhangjiakou Fm., Late Jurassic, Liaoning, China; Qingtujin Gr., Middle Jurassic, China; Wanbao Fm., Middle Jurassic, Inner Mongolia, China; Longfengshan Fm., Middle Jurassic, Gansu, China; Xintiangou Fm., Middle Jurassic, Guizhou, China	Zhang et al., 1976; Feng, 1978; Lu, 1995; Huang et al., 1998; Shen et al., 2003; Shen, 2004; Bao et al., 2011; Zhang et al., 2012; Zhang et al., 2013; Zhang et al., 2016. [2,113,143,144,145,146,147,148,149,150]
*Euestheria* aff. *ziliujingensis*	Xiaodonggou Fm., Middle Jurassic, Liaoning, Jilin, China; Anning Fm., Tuodian Fm., Late Jurassic, Yunnan, China	Fan, 1996; Cheng et al., 2004. [151,152]
*Triglypt haifanggouensis* Liao, 2017 (=*Euestheria haifanggouensis* Chen, 1976)	Haifanggou Fm., Middle Jurassic, Liaoning, China; Zhanghe Fm., Middle Jurassic, Yunan, China; Wangjiashan Fm., Middle Jurassic, Gansu, China; Shaximiao Fm., Middle Jurassic, Guizhou, China; Dongyingfang Fm., Middle Jurassic, Liaoning, China; Luoao Fm., Middle Jurassic, Jiangxi China; Jiulongshan Fm., Middle Jurassic, Inner Mongolia, China; Wanbao Fm., Middle Jurassic, Inner Mongolia, China; Zhangjiakou Fm., Early Jurassic, Inner Mongolia, China	Zhang et al., 1976; Shen et al., 2003; Liao et al., 2017; Feng, 1978; Li, 1990; Huang et al., 1998; Bao et al., 2011; Zhang et al., 2013. [2,139,143,144,145,147,149,153]
*Euestheria* aff. *haifanggouensis*	Xiaodonggou Fm., Middle Jurassic, Liaoning, Jilin, China; Xintiangou Fm., Middle Jurassic, Guizhou, China	Fan, 1996; Zhang et al., 2016. [150,151]
*Euestheria* cf. *haifanggouensis*	Hadataolegai Fm., Middle Jurassic, Jilin, China	Tan et al., 2019. [154]
*Euestheria rotunda* Zhang, 1976	Shaximiao Fm., Middle Jurassic, Sichuan, China; Qingtujin Gr., Middle Jurassic, China	Zhang et al., 1976; Zhang et al., 2016; Shen, 2004. [2,146,150]
*Euestheria* ? *subquadrata* Chen, 1976	Wangjiashan Fm., Middle Jurassic, Gansu, China	Zhang et al., 1976. [2]
*Euestheria changtanensis* Shen,1976	Ziliujing Fm., Middle Jurassic, Sichuan, China	Zhang et al., 1976. [2]
*Euestheria elegans* Shen, 1976	Guizhou Gr., Middle Jurassic, Hubei, China	Zhang et al., 1976. [2]
*Euestheria* cf. *elegans*	Sangonghe Fm., Early Jurassic, Xinjiang, China	Fu, 1998. [143]
*Euestheria orientalis* Shen, 1976	Guizhou Gr., Middle Jurassic, Hubei, China	Zhang et al., 1976. [2]
*Euestheria jingyuanensis* Chen, 1976	Wangjiashan Fm., Middle Jurassic, Gansu, China; Toutunhe Fm., Middle Jurassic, Xinjiang, China; Hadataolegai Fm., Middle Jurassic, Jilin, China; Jiulongshan Fm., Middle Jurassic, Inner Mongolia, China; Shaximiao Fm., Middle Jurassic, Guizhou, Sichuan, China; Tuodian Fm., Late Jurassic, Yunnan, China; Xinhe Fm., Middle Jurassic, Gansu, China	Zhang et al., 1976; Feng, 1978; Lu, 1995; Shen et al., 2003; Cheng et al., 2004; Peng et al., 2016; Gao et al., 2017; Tan et al., 2019. [2,113,140,143,152,154,155,156]
*Euestheria* cf. *jingyuanensis*	Qingtujin Gr., Middle Jurassic, China	Shen, 2004. [146]
*Euestheria fabiformis* Chen, 1976	Wangjiashan Fm., Middle Jurassic, Gansu, China; Xinhe Fm., Middle Jurassic, Gansu, China	Zhang et al., 1976; Peng et al., 2016. [2,156]
*Euestheria* aff. *fabiformis*	Qingtujin Gr., Middle Jurassic, China	Shen, 2004. [146]
*Euestheria yanjiawanensis* Chen, 1976	Xiashaximiao Fm., Middle Jurassic, Sichuan, China; Wangjiashan Fm., Middle Jurassic, Gansu, China; Anning Fm., Late Jurassic, Yunnan, China; Qingtujin Gr., Middle Jurassic, China; Zhangping Fm., Middle Jurassic, Fujian, China	Zhang et al., 1976; Liang, 1998; Cheng et al., 2004; Shen, 2004; Gao et al., 2017. [2,135,147,153,156]
*Euestheria* ? cf. *yanjiawanensis*	Laohugou Fm., Early Jurassic, Liaoning, China	Li et al., 1982; Shen & Chang, 1993. [40,141]
*Euestheria complanata* Chen, 1976	Guangyuan Gr., Middle Jurassic, Sichuan, China; Zhangping Fm., Middle Jurassic, Fujian, China; Dongyingfang Fm., Middle Jurassic, Liaoning, China; Qingtujin Gr., Middle Jurassic, Inner Mongolia, China; Longfengshan Fm., Middle Jurassic, Gansu, China; Shaximiao Fm., Middle Jurassic, Sichuan, China	Zhang et al., 1976; Cao et al., 1989; Liang, 1998; Li, 1990; Shen, 2004; Zhang et al., 2013; Gao et al., 2017. [2,135,136,139,146,149,156]
*Euestheria* ? cf. *complanata*	Anning Fm., Late Jurassic, Yunnan, China	Li et al., 1982; Cheng et al., 2004. [141,152]
*Euestheria xiazhuangensis* Chen, 1976	Zhanghe Fm., Middle Jurassic, Yunnan, China	Zhang et al., 1976. [2]
*Euestheria manzhuangensis* Chen, 1976	Huakaizuo Fm., Middle Jurassic, Yunnan, China; Qiakemake Fm., Middle Jurassic, Xinjiang, China	Zhang et al., 1976; Liu, 1990. [2,157]
*Euestheria* cf. *manzhuangensis*	Anning Fm., Late Jurassic, Yunnan, China	Cheng et al., 2004. [152]
*Euestheria exilis* Chen, 1976	Huakaizuo Fm., Middle Jurassic, Yunnan, China	Zhang et al., 1976. [2]
*Euestheria* ? *yangbiensis* Chen, 1976	Huakaizuo Fm., Middle Jurassic, Yunnan, China	Zhang et al., 1976. [2]
*Euestheria* cf. *yangbiensis*	Tuoman Fm., Late Jurassic, Yunnan, China	Cheng et al., 2004. [152]
*Euestheria* ? *batangensis* Chen, 1976	Late Triassic, Sichuan, China	Zhang et al., 1976. [2]
*Euestheria* ? *favosa* Chen, 1976	Dalazi Fm., Early Cretaceous, Jilin, China	Zhang et al., 1976. [2]
*Euestheria* ? *ambiqua* Zhang & Chen, 1976	Nenjiang Fm., Early Cretaceous, Heilongjiang, China	Zhang et al., 1976. [2]
*Euestheria datongensis* Zhang, 1976	Yungang Fm., Middle Jurassic, Shanxi, China	Zhang et al., 1976. [2]
*Euestheria* ? *nanchuanensis* Chen, 1976	Shangshaximiao Fm., Middle Jurassic, Sichuan, China	Zhang et al., 1976; Gao et al., 2017. [2,156]
*Euestheria fuyuanensis* Chen,1976	Kayitou Fm., Early Triassic, Guizhou, Yunnan, China	Zhang et al., 1976; Scholze et al., 2020. [2,80]
*Euestheria wuzaoensis* Chen & shen, 1978	Wuzao Fm., Late Triassic, Zhejiang, China	Chen, 1978. [158]
*Euestheria trinanguliformis* Duan, 1978	Badong Fm., Middle Triassic, Sichuan, China	Duan, 1978. [159]
*Euestheria nanxiensis* Duan, 1978	Xujiahe Fm., Late Triassic, Sichuan, China	Duan, 1978. [159]
*Euestheria dongyuemiaoensis* Duan, 1978	Ziliujing Fm., Middle Jurassic, Sichuan, China	Duan, 1978. [159]
*Euestheria zizhongensis* Duan, 1978	Ziliujing Fm., Middle Jurassic, Sichuan, China	Duan, 1978. [159]
*Euestheria jiangyouensis* Duan, 1978	Xiashaximiao Fm., Middle Jurassic, Sichuan, China	Duan, 1978. [159]
*Euestheria bijieensis* Feng, 1978	Shaximiao Fm., Middle Jurassic, Guizhou, China	Feng, 1978. [143]
*Euestheria* ? *xiangshuiensis* Feng, 1978	Shaximiao Fm., Middle Jurassic, Guizhou, China	Feng, 1978. [143]
*Euestheria* ? *oertlii* Kozur, 1980	Bernburg Fm., Early Triassic, Germany	Kozur, 1980. [137]
*Euestheria tongchuangensis* Liu, 1980	Tongchuan Fm., Middle Triassic, Shaanxi, China	Wang & Liu, 1980. [39]
*Euestheria gibba* Liu, 1980	Tongchuan Fm., Middle Triassic, Shaanxi, China	Wang & Liu, 1980. [39]
*Euestheria huanglongensis* Liu, 1980	Tongchuan Fm., Middle Triassic, Shaanxi, China	Wang & Liu, 1980. [39]
*Euestheria celeta* Liu, 1980	Tongchuan Fm., Middle Triassic, Shaanxi, China	Wang & Liu, 1980. [39]
*Euestheria jinsuoguanensis* Liu, 1980	Tongchuan Fm., Middle Triassic, Shaanxi, China	Wang & Liu, 1980. [39]
*Euestheria hanchengensis* Liu, 1980	TongchuanFm., Middle Triassic, Shaanxi, China	Wang & Liu, 1980. [39]
*Euestheria deforma* Liu, 1980	Yanchang Gr., Late Triassic, Shaanxi, China	Wang & Liu, 1980. [39]
*Euestheria shensiensis* Liu, 1980	Yanchang Gr., Late Triassic, Shaanxi, China	Wang & Liu, 1980. [39]
*Euestheria changhangouensis* Wu, 1980	Changhangou Fm., Middle Jurassic, Inner Mongolia, China	Wu, 1980. [160]
*Euestheria* cf. *changhangouensis*	Mawa Fm., Middle Jurassic, Henan, China	Hu, 1991. [161]
*Euestheria shiguaiziensis* Wu, 1980	Changhangou Fm., Middle Jurassic, Inner Mongolia, China	Wu, 1980. [160]
*Euestheria* cf. *shiguaiziensis*	Mawa Fm., Middle Jurassic, Henan, China	Hu, 1991. [161]
*Euestheria jiangdaensis* Chen & Shen, 1981	Jiapeila Fm., Late Triassic, Tibet, China	Chen & Shen, 1981. [128]
*Euestheria deyiensis* Chen & Shen, 1981	Jiapeila Fm., Late Triassic, Tibet, China	Chen & Shen, 1981. [128]
*Euestheria waxianensis* Chen & Shen, 1981	Shangshaximiao Fm., Middle Jurassic, Sichuan, China	Chen & Shen, 1981. [128]
*Euestheria shandongensis* Chen, 1982	Wennan Fm., Early Jurassic, Shandong, China.	Chen, 1982. [114]
*Euestheria rongxianensis* Xu, 1982	Xiashaximiao Fm., Middle Jurassic, Sichuan, China	Xu, 1982. [162]
*Euestheria truempyi* Shen, 2002 (=*Magniestheria truempyi* Kozur & Seidel, 1983)	Early Triassic, Germany	Kozur & Seidel, 1983; Shen, 2002. [76,163]
*Euestheria jimsarensis* Wei, 1984	Huangshanjie Fm., Late Triassic, Xinjiang, China	Wei, 1984. [73]
*Euestheria stockleyi* Tasch, 1984	Late Triassic, Africa	Tasch, 1984. [164]
*Euestheria* ? *chaohuensis* Shen, 1985	Hanshan Fm., Middle Jurassic, Anhui, China	Lu et al., 1985. [165]
*Euestheria* ? *pengzhuanensis* Shen, 1985	Hanshan Fm., Middle Jurassic, Anhui, China	Lu et al., 1985. [165]
*Euestheria taschi* Vallati, 1986	Cañadón Asfalto Fm., Chubut, Argentina	Vallati, 1986; Gallego, 2010. [96,166]
*Euestheria thabaningensis* Tasch, 1987	Late Triassic, Africa	Tasch, 1987. [19]
*Euestheria triassibrevis* Tasch, 1987	Maji ya Chumvi Fm., Early Triassic, Africa	Tasch, 1987. [19]
*Triglypta luanpingensis* Liao, 2017 (=*Euestheria luanpingensis* Shen & Niu, 1987)	Jiulongshan Fm., Middle Jurassic, Hebei, Inner Mongolia, China	Zhang et al., 1987; Shen et al., 2003; Liao et al., 2017. [145,153,166]
*Euestheria dakongensis* Cao, 1987	Lishan Fm., Early Jurassic, Fujian, China; Xiaoping Fm., Late Triassic, Guangdong, China	Cao, 1986; Liang, 1998; Zhang & Lin, 2000. [35,135,167]
*Euestheria multicostata* Geyer, 1987	Sanford Fm., Amstadt Fm., Late Triassic, Thuringia, Germany	Kozur & Weems, 2007. [36]
*Euestheria lashlyensis* Tasch, 1987	Middle–Late Devonian, Lashly Mountains, Antarctica	Tasch, 1987. [19]
*Euestheria ritchiei* Tasch, 1987	Late Devonian, South Victoria Land, Antarctica	Tasch, 1987. [19]
*Euestheria juravariabalis* Tasch, 1987	Early Jurassic, Mauger Nunatak, Antarctica	Tasch, 1987. [19]
*Euestheria bearmorensis* Tasch, 1987	Early Jurassic, BH Station, Blizzard Heights, Antarctica	Tasch, 1987. [19]
*Euestheria ellioti* Tasch, 1987	Early Jurassic, BH Station, Blizzard Heights, Antarctica	Tasch, 1987. [19]
*Euestheria juracircularis* Tasch, 1987	Early Jurassic, BH Station, Blizzard Heights, Antarctica	Tasch, 1987. [19]
*Euestheria formavariabalis* Tasch, 1987	Early Jurassic, BH Station, Blizzard Heights, Antarctica	Tasch, 1987. [19]
*Euestheria ichthystromatos* Tasch, 1987	Early Jurassic, Station 0, Storm Peak, Antarctica	Tasch, 1987. [19]
*Euestheria crustapatulis* Tasch, 1987	Early Jurassic, Storm Peak, Antarctica	Tasch, 1987. [19]
*Euestheria rhadinis* Tasch, 1987	Early Jurassic, Storm Peak, Antarctica	Tasch, 1987. [19]
*Euestheria transantarctensis* Tasch, 1987	Early Jurassic, Storm Peak, Antarctica	Tasch, 1987. [19]
*Euestheria castaneus* Tasch, 1987	Early Jurassic, Storm Peak, Antarctica	Tasch, 1987. [19]
*Euestheria talenti* Tasch, 1987	Devonian, Victoria, Australia	Tasch, 1987. [19]
*Euestheria abaetensis* Tasch, 1987 (=*Platyestheria* abaetensis Cardoso, 1971)	Quirico Fm., Early Cretaceous, Sanfranciscana Basin, Brazil	Tasch, 1987; Carvalho, 1993; Cardoso, 1971. [19,168,169]
*Euestheria crustapatulus* Tasch, 1987	Ferrar Gr., Storm Peak, Antarctica	Tasch, 1987; Carvalho, 1993. [19,168]
*Euestheria thabaningensis* Tasch, 1987	Late Triassic, Lesotho	Tasch, 1987. [19]
*Euestheria triassibrevis* Tasch, 1987	Narrabeen Gr., Early Triassic, Port Hacking, Australia	Tasch, 1987. [19]
*Euestheria basbatiliensis* Tasch, 1987	Panchet Fm., Early Triassic, Raniganj Coal Field, India	Tasch, 1987. [19]
*Euestheria raniganjis* Tasch, 1987	Panchet Fm., Early Triassic, Raniganj Coal Field, India	Tasch, 1987. [19]
*Euestheria dualis* Tasch, 1987	Panchet Fm., Early Triassic, Raniganj Coal Field, India	Tasch, 1987. [19]
*Euestheria crustabundis* Tasch, 1987	Kotá Fm., Early Jurassic, India	Tasch, 1987. [19]
*Euestheria lefranci* Tasch, 1987	Late Cretaceous, Algeria, Africa	Tasch, 1987. [19]
*Euestheria aricensis* (Jones) Tasch, 1987	Permian or Triassic, Arica, Arequipa, Peru	Tasch, 1987. [19]
*Euestheria* cf. *aricensis*	Permian, Chile	Tasch, 1987. [19]
*Euestheria nitida* Wu, 1991	Ermaying Fm., Middle Triassic, Shaanxi, China	Wu, 1991. [120]
*Euestheria brevielliptica* Wu, 1991	Ermaying Fm., Middle Triassic, Shaanxi, China	Wu, 1991. [120]
*Punctatestheria trotternishensis* Zhang, 2017 (=*Euestheria trotternishensis* Chen & Hudson, 1991)	Lealt Shale Fm., Middle Jurassic, Skye, Scotland	Chen & Hudson, 1991; Zhang et al., 2017. [170,171]
*Euestheria falconeri* Gallego, 1993	Yaguarı Fm., Late Permian, Uruguay	Gallego et al., 1993. [172]
*Euestheria rocablanquensis* Gallego, 1994	Roca Blanca Fm., Early–Middle Jurassic, Santa Cruz, Argentina	Gallego, 1994. [173]
*Euestheria covacevichi* Gallego, 1994	Peine Fm., Late Permian, Antofagasta, Chile	Gallego & Breitkreuz, 1994. [174]
*Euestheria santamariensis* Gallego, 1996	Santa Maria Fm., Middle Triassic, Brazil	Gallego, 1996. [52]
*Euestheria pricei* Gallego, 1999	Castellanos Fm., Early Cretaceous, Uruguay	Gallego et al., 1999. [175]
*Euestheria feysi* Vannier, 2003	Carboniferous, Saône-et-Loire, France	Vannier et al., 2003. [176]
*Euestheria martinsnetoi* Gallego, 2004	Ria Mendzo Fm., Middle Triassic, Mendoza, Argentina	Gallego et al., 2004. [177]
*Euestheria urengoic* Chunikhin, 2004	Late Triassic, Western Siberia, Russia	Chunikhin, 2004. [178]
*Euestheria duqinshanensis* Shen, 2004	Qingtujin Gr., Middle Jurassic, Inner Mongolia, China	Shen, 2004. [146]
*Euestheria acampestira* Bishop, 2010	Early–Middle Triassic, Aranbanga Volcanics Gr., Queensland, Australia	Bishop, 2010. [179]
*Euestheria sanrensis* Ghosh, 2011	Pali Fm., Permian–Early Jurassic, Jharkhand, India	Ghosh, 2011. [5]
*Euestheria menendezi* Gallego & Tassi, 2015	Potrerillos Fm., Middle Triassic, Mendoza, Argentina	Tassi et al., 2015. [41]
*Euestheria kozuri* Geyer, 2018	Hassberge Fm., Late Triassic, Franconia, Germany	Geyer & Kelber, 2018. [21]
*Pseudestherites musacchioi* Gallego, 2004 (=*Euestheria* sp.)	La Amarga Fm., Early Cretaceous, Neuquén, Argentina	Musacchio, 1970; Gallego & Shen, 2004. [180,181]
*Euestheria* sp. undet	New South Wales, Australia	Tasch, 1987. [19]
*Euestheria* sp.	Longtan Fm., Late Permian, Zhejiang, China	Tang et al., 1980. [182]
*Euestheria* sp.	Cumnock Fm., Late Triassic, Goldston Quadrangle, Chatham County, USA	Kozur & Weems, 2010. [4]
*Euestheria* sp.	Sangonghe Fm., Early Jurassic, Xinjiang, China	Fu, 1998. [142]
*Euestheria* spp.	Middle Permian, Kansas, USA	Tasch, 1958. [105]
*Euestheria* sp.	Albert Fm., New Brunswick, Canada	Greiner, 1974. [110]
*Euestheria* sp.	Anning Fm., Late Jurassic, Yunnan, China	Cheng et al., 2004. [152]
*Euestheria* sp.	Canadon Asfalto Fm., Jurassic, Patagonia, South America	Tasch, 1970. [124]
*Euestheria* spp.	Ershierzhan Fm., Middle Jurassic, Heilongjiang, China	Wang, 1985. [183]
*Euestheria* ? sp.	Hassberge Fm., Late Triassic, Eltmann, Franconia, Germany	Geyer & Kelber, 2018. [21]
*Euestheria* spp.	Huangmaqing Fm., Late Triassic, Jiangsu, China	Wu, 1980. [184]
*Euestheria* sp.	Jurassic, Argentina	Vallati, 1986. [185]
*Euestheria* spp.	Kayitou Fm., Early Triassic, Guizhou, China	Chu et al., 2013. [78]
*Euestheria* sp.	Laohugou Fm., Late Triassic, Liaoning, China	Zhang & Dong, 1982. [186]
*Euestheria* sp.	Late Triassic, Germany	Barth & Kozur et al., 2011. [187]
*Euestheria* sp.	Tuckahoe Fm., Late Triassic, North Carolina, USA	Kozur & Weems, 2010. [4]
*Euestheria* sp.	Longfengshan Fm., Middle Jurassic, Gansu, China	Zhang et al., 2013. [149]
*Euestheria* sp.	Longfenshan Fm., Middle Jurassic, Gansu, China	Li et al., 1982. [141]
*Euestheria* sp.	Longtan Fm., Late Permian, Zhejiang, China	Tang et al., 1980. [182]
*Euestheria* sp.	Manantial Pelado Fm., Middle Jurassic, Chubut, Argentina	Gallego, 2010. [188]
*Euestheria* sp.	Moenave Fm., Early Jurassic, Utah, USA	Milner, 2006. [189]
*Euestheria* sp.	Pastos Bons Fm., Late Jurassic, Piaui, Brazil	Tasch, 1987. [19]
*Euestheria* spp.	Rio do Rasto Fm., Late Permian, Santa Catarina, Brazil	Tasch, 1987. [19]
*Euestheria* spp.	Sangonghe Fm., Early Jurassic, Xinjiang, China	Lu, 1995. [113]
*Euestheria* sp.	Solling Fm., Triassic, Bavaria, Germany	Kozur & Mock, 1993. [44]
*Euestheria* sp.	Tiaojishan Fm., Middle Jurassic, Liaoning, China	Gao et al., 2017. [156]
*Euestheria* sp.	Early Jurassic, Storm Peak, Antarctica	Tasch, 1987. [19]
*Euestheria* sp.	Wenbinshan Fm., Late Triassic, Fujian, China	Liang, 1998. [135]
*Euestheria* sp.	Xiaodonggou Fm., Middle Jurassic, Liaoning, Jilin, China	Fan, 1996. [151]
*Euestheria* sp.	Xingfuzhilu Fm., Early Triassic, Inner Mongolia, China	Zheng et al., 2013. [190]
*Euestheria* sp.	Xintiangou Fm., Middle Jurassic, Guizhou, China	Zhang et al., 2016. [150]
*Euestheria* ? sp.	Xishanyao Fm., Middle Jurassic, Xinjiang, China	Lu, 1995. [113]
*Euestheria* spp.	Xujiashan Fm., Middle Triassic, Jiangsu, China	Min et al., 1981. [138]
*Euestheria* spp.	Santa Maria Fm., Middle Triassic, Brazil	Katoo, 1971. [26]
*Euestheria* sp.	Peine Fm., Late Permian, Antofagasta, Chile	Gallego & Breitkreuz, 1994. [174]
*Euestheria* sp.	Roca Blanca Fm., Early–Middle Jurassic, Santa Cruz, Argentina	Gallego, 1994. [173]
*Euestheria* sp.	Ischichuca Fm, Middle–Late Triassic, La Rioja, Argentina	Gallego et al., 2001. [51]
*Euestheria* sp.	Late Triassic, Western Siberia, Russia	Chunikhin, 2004. [178]
*Euestheria* sp.	Late Cretaceous, Africa	Mouta & Marlière, 1950. [90]
*Euestheria* sp.	Badaowan Fm., Early Jurassic, Xinjiang, China	Shen, 2003. [140]
*Euestheria* sp.	Houjiatun Fm., Late Jurassic, Liaoning, China	Shen, 2003. [140]
*Euestheria* ? sp.	Shanglufeng Fm., Middle Jurassic, Yunnan, China	Zhang et al., 1976. [2]

## Data Availability

No new data were created or analyzed in this study. Data sharing is not applicable to this article.
